# Association between Anxiety and Vascular Dementia Risk: New Evidence and an Updated Meta-Analysis

**DOI:** 10.3390/jcm9051368

**Published:** 2020-05-06

**Authors:** Javier Santabárbara, Darren M. Lipnicki, Beatriz Olaya, Beatriz Villagrasa, Patricia Gracia-García, Juan Bueno-Notivol, Antonio Lobo, Raúl López-Antón

**Affiliations:** 1 Department of Preventive Medicine and Public Health, Universidad de Zaragoza, 50009 Zaragoza, Spain; jsantabarbara@unizar.es; 2 Instituto de Investigación Sanitaria de Aragón (IIS Aragón), 50009 Zaragoza, Spain; alobo@unizar.es (A.L.); rlanton@unizar.es (R.L.-A.); 3 Centro de Investigación Biomédica en Red de Salud Mental (CIBERSAM), Ministry of Science and Innovation, 28029 Madrid, Spain; 4 Centre for Healthy Brain Ageing, School of Psychiatry, University of New South Wales Medicine, Randwick, NSW 2052, Australia; d.lipnicki@unsw.edu.au; 5 Research, Innovation and Teaching Unit, Parc Sanitari Sant Joan de Déu, Universitat de Barcelona, 08830 Sant Boi de Llobregat, Spain; 6 Psychogeriatry, CASM Benito Menni, 08830 Sant Boi de Llobregat, Spain; beavibla@gmail.com; 7 Psychiatry Service, Hospital Universitario Miguel Servet, 50009 Zaragoza, Spain; pgraciagarcia@yahoo.es (P.G.-G.); elecrijuan@hotmail.com (J.B.-N.); 8 Department of Medicine and Psychiatry, Universidad de Zaragoza, 50009 Zaragoza, Spain; 9 Department of Psychology and Sociology, Universidad de Zaragoza, 50009 Zaragoza, Spain

**Keywords:** anxiety, vascular dementia, risk factor, ZARADEMP, meta-analysis

## Abstract

The association between anxiety and vascular dementia (VaD) is unclear. We aimed to reliably estimate the association between anxiety and VaD risk using meta-analysis to pool new results from a large community-based cohort (Zaragoza Dementia and Depression (ZARADEMP) study) and results from previous studies. ZARADEMP participants (*n* = 4057) free of dementia were followed up on for up to 12 years. Cases and subcases of anxiety were determined at baseline. A panel of four psychiatrists diagnosed incident cases of VaD by consensus. We searched for similar studies published up to October 2019 using PubMed and Web of Science. Observational studies reporting associations between anxiety and VaD risk, and adjusting at least for age, were selected. Odds ratios (ORs) from each study were combined using fixed-effects models. In the ZARADEMP study, the risk of VaD was 1.41 times higher among individuals with anxiety (95% CI: 0.75–2.68) compared with non-cases (*p* = 0.288). Pooling this result with results from two previous studies yielded an OR of 1.65 (95% CI: 1.07–2.53; *p* = 0.022). These findings indicate that anxiety is associated with an increased risk of VaD. Taking into account that anxiety is commonly observed in the elderly, treating and preventing it might reduce the prevalence and incidence of VaD. However, whether anxiety is a cause of a prodrome of VaD is still unknown, and future research is needed to clarify this.

## 1. Introduction

Dementia prevalence rates increase in parallel to population age. In 2018, around 50 million people globally suffered from dementia, but with population aging, this number is estimated to be three times higher by 2050 [1]. Dementia is a severe neurological condition, with considerable social, familiar, and economic consequences. As dementia is currently without a cure, the design of preventive strategies to reduce the risk and delay its onset is a public health priority [2]. To this end, identifying the risk factors associated with dementia and the magnitude of their effects is essential. Systematic review and meta-analysis (MA) are among the best approaches to help to reach this goal [3].

Anxiety disorders are one of the most common mental disorders [4] and have been recognized as potential risk factors for all-cause dementia and Alzheimer’s disease (AD). This has been demonstrated by our recent meta-analyses that found that people with an anxiety disorder have a 29% and 45% greater risk of suffering from all-cause dementia and AD, respectively [5,6]. However, the association between anxiety and vascular dementia (VaD), which accounts for 20% of age-related dementias [7], is less clear. A recent systematic review and MA detailed three studies that individually found no significant association between anxiety and VaD [8]. However, pooled results from the two studies reporting odds ratios showed that anxiety was associated with an 88% increased risk for VaD [8]. In the current paper, we investigate whether anxiety is associated with VaD in a large, population-based cohort not featured in the prior MA. We also include the results from this cohort in a larger MA to better estimate the magnitude of association between anxiety and incident VaD.

## 2. Methods

This work follows the STrengthening the Reporting of OBservational studies in Epidemiology (STROBE) [9] and Statistical Analyses and Methods in the Published Literature (SAMPL) [10] guidelines for reporting observational studies in epidemiology and statistics, respectively. The MA part was conducted in accordance with the Preferred Reporting Items for Systematic Reviews and Meta-Analyses (PRISMA) guidelines for reporting systematic reviews and meta-analysis [11] (Supplementary Table S1).

### 2.1. Sample and Procedure

We included data from the Zaragoza Dementia and Depression (ZARADEMP) Project, a longitudinal, population-based study aimed at determining the incidence of and risk factors for somatic and psychiatric disorders among people aged ≥ 55 years. The study was approved by the Ethics Committee of the Institutional Review Board in our institutions (CEICA) in accordance with Spanish Law. Written informed consent was obtained from all participants.

The methodology of ZARADEMP has been previously described [12,13,14,15]. Briefly, a random sample of community-dwelling people aged 55 years or older was proportionally drawn by age and sex from the census list of Zaragoza (Spain). The first assessment took place in 1994, and a total of 4803 individuals were interviewed with a refusal rate of 20.5%. The present study includes results from the baseline study (wave I) and three follow-up waves (waves II, III, and IV). Individuals identified as cases or subcases of dementia (according to the GSM) at baseline were excluded from the analysis (*n* = 746).

We followed a two-phase screening procedure in each wave. The validated Spanish versions of the following questionnaires were used: Mini-Mental Status Examination (MMSE) [16] and Geriatric Mental State (GMS), which includes a semistructured standardized clinical interview to assess mental state of older adults. These data were then used by the AGECAT (Automated Geriatric Examination for Computer Assisted Taxonomy) system, which identifies the presence of several psychiatric diagnoses [17]; the History and Aetiology Schedule (HAS) [18] to collect medical and psychiatric history data; Katz’s index for basic activities of daily living (bADLs) [19] and the Lawton and Brody scale for instrumental activities (iADLs) [20]; and the European Studies of Dementia (EURODEM) risk factors questionnaire [21] to assess the presence of medical conditions.

### 2.2. Assessment and Diagnosis of Vascular Dementia

A clinician (psychiatrist) made an initial diagnosis of VaD using both Diagnostic and Statistical Manual, Fourth Edition (DSM-IV) [22] and International Classification of Diseases 10th edition (ICD-10) [23] criteria, which was finally confirmed by consensus. The ZARADEMP interview generates enough information to diagnose in accordance with all these criteria [24]. Agreement had to be reached by at least three out of four psychiatrists. The validity of the diagnostic process has been previously reported [9]. To ensure the accuracy of the diagnosis process, some diagnosed cases were invited to participate in a hospital work-up, where neuroimaging studies, a neuropsychological battery and the Hachinski scale criteria were applied [25]. We found a 95.8% agreement on the diagnosis of dementia, and 87.5% agreement on the subtype of dementia.

### 2.3. Assessment and Diagnosis of Anxiety

The GMS–AGECAT system was used to stablish diagnoses of anxiety [26]. This computerized algorithm compares syndrome clusters (e.g., dementia, depression, anxiety) to reach a final diagnosis, recorded as either a “subcase” (“subsyndromal”, confidence levels 1 and 2) or a “case” of anxiety (confidence levels ≥ 3). If AGECAT score is ≥ 3, a subject is considered as “case” of anxiety (clinically relevant anxiety that requires clinical intervention). If the score is 1 or 2, that subject is considered as a “subcase” of anxiety (subsyndromal or mild anxiety), whereas a score of 0 is considered as “non-case”. Previous studies have stablished the validity of this method, with an overall agreement of 89% between psychiatric diagnosis and the classification made by the system, and a Cohen’s kappa value of 0.74 [17].

In a previous study conducted with ZARADEMP data [14], cases and subcases of anxiety were separately studied to determine the association with all-cause dementia (including AD and VaD). However, the present MA focused only on the VaD subtype. Due to the low number of incident cases of VaD in the ZARADEMP study, and in order to increase the statistical power, cases and subcases were grouped into a single anxiety category. Additionally, symptoms associated with both anxiety and subsyndromal anxiety symptoms cause significant functional impairment [27,28,29,30], thus making the use of the merged category for anxiety appropriate.

### 2.4. Covariates

The analysis included the following covariates at baseline: sociodemographic variables (age, sex, educational level, marital status, and living alone), chronic conditions (vascular disease, diabetes, obesity, and high blood pressure), health status, presence of depression, and cognitive function (measured with the MMSE) [14]. Additionally, we included vascular risk factors (smoking, statin use, body mass index, and alcohol consumption) obtained with the EURODEM risk factors questionnaire [21].

### 2.5. Statistical Analysis

Differences between baseline characteristics according to anxiety status were assessed using two-tailed chi-square tests for categorical data, and using *t* tests for variables with an approximately normal distribution.

Multivariate logistic regression analysis was performed to calculate the risk of VaD according to anxiety status. We used two models, with the first adjusted for sociodemographic characteristics, and the second adjusted for sociodemographic characteristics, medical and vascular risk factors, health status, depression, cognitive status, and follow-up duration. We explored two-way interactions between the independent variables, but no significant interactions were found.

### 2.6. Meta-Analysis

Two researchers (BV and JBN) searched for all cohort or case-control studies reporting the association between anxiety and risk of VaD published between January 2018 and October 2019 using MEDLINE and Web of Science, since the aim was to update the previous systematic review [8] published in 2018, adding to it all potential papers published after Becker et al.’s [8] work. In brief, the search strategy included the following terms: (anxiety AND Vascular Dementia AND (cohort studies OR longitudinal OR case-control study)). Medical subject headings and free text were used and only studies written in English. Supplementary Table S2 displays the search strategy. Any disagreement was resolved by consensus with a third and fourth reviewer (PGG and RLA).

Studies were included if: (1) It was a cohort or case-control study; (2) anxiety was assessed at baseline; (3) dementia or mild cognitive impairment (MCI) were absent at baseline; (4) they reported the link between anxiety and VaD incidence; and (5) they included a summary estimate, i.e., relative risk (RR), odds ratio (OR), hazard ratio (HR) or subdistribution hazard ratio (SHR), with their confidence intervals. We excluded studies focusing on MCI samples, review articles and MAs, or those not reporting original, peer-reviewed findings.

A predesigned data extraction form was used to extract information on the following: country, sample size, prevalent rates of anxiety, incident cases of VaD, proportion of females, average age, instruments used to assess anxiety, clinical criteria used for VaD diagnosis or assessment, other adjusting covariates, follow-up period, and adjusted risk estimates.

We used the Newcastle–Ottawa scale (NOS) for cohort and case-control studies to assess the quality of the studies included [31]. This nine-point scale evaluates the risk of bias based on the population selection, comparability and outcome. Scores of 0–3, 4–6, and 7–9 indicate low, moderate, and high quality, respectively. Two authors (JS and BO) evaluated the quality of the included studies, and if there was some disagreement, a consensus decision was reached.

We used ORs from fully adjusted models as a measure of association across studies to calculate the pooled risk estimates. The effect size (Cohen’s *d*) for overall ORs and their confidence intervals was calculated according to Sánchez-Meca et al. [32]. Values of ~0.2 were considered as “small”, ~0.5 as “medium”, and ~0.8 as “large” [33].

The Hedges *Q* statistic was reported to check heterogeneity across studies, with a statistical significance set at *p* < 0.10. Following the recommendations for a small number of studies [34], the *I^2^* statistic [35] and 95% confidence interval were also used to quantify heterogeneity. *I^2^* values between 25% and 50% are considered as low, moderate for 50−75%, and high for 75% [35]. Heterogeneity of effects between studies occurs when the differences in results for the same exposure–disease association cannot be fully explained by sampling variation. Sources of heterogeneity can include differences in study design or in demographic characteristics. We conducted a sensitivity analysis to determine the influence of each individual study on the overall result by omitting them one by one. Since a funnel plot could be misleading when there are less than 10 studies in an MA [36], publication bias was determined with the fail-safe *N* value [37]. A fail-safe number (N) indicates the number of nonsignificant, unpublished (or missing) studies that would be needed to be added to an MA to reduce an overall statistically significant observed result to nonsignificance. There is confidence in the summary conclusions if this number is large relative to the number of observed studies [37].

Statistical analyses were conducted by JS and run with STATA statistical software (version 10.0; College Station, TX, USA) and R version 3.6.2 (R Core Team, Vienna, Austria, 2019).

## 3. Results

### 3.1. Association between Anxiety and VaD in the ZARADEMP Sample

The sample included 4057 participants free of dementia during the 12 years of follow-up. Of all included participants, 1763 (42.8%) were classified as anxiety ‘cases’ or ‘subcases’, and 2321 (57.2%) were classified as ‘non-cases’. Sociodemographic characteristics at baseline according to anxiety status have been described elsewhere [13]. Briefly, participants who were considered cases and subcases of anxiety were more likely to be female, have depression, report poor health status, and present disabilities for instrumental ADLs, compared with non-cases.

Table 1 displays sociodemographic and clinical characteristics at baseline for participants who did not develop VaD during the follow-up period (*n* = 4013) and for those who did (*n* = 44). The incident VaD group was significantly older, more likely to be divorced, separated or widowed, and have hypertension, vascular disease and worse cognitive function.

The mean follow-up time was 7.5 (SD = 4.2) years, with a maximum of 12 years. Forty-four participants (1.1%) developed VaD during follow-up. In the multivariate analysis, the risk of VaD was 41% higher among anxiety cases compared with non-cases but did not reach statistical significance (Table 2, model 2).

### 3.2. Meta-Analysis

#### 3.2.1. Study Selection

Supplementary Figure S1 shows the flowchart used for the strategy and process of the literature search and selection of studies. A total of 1088 potential records were yielded in the first search, from which 322 duplicates were removed. After reading the titles and abstracts of the remaining 766 articles, 751 were excluded. We then read in full the 15 remaining articles, and all were excluded (2 were not cohort/case studies; 13 did not address the association between anxiety and VaD risk). Thus, no study after the publication of the systematic review by Becker et al. [8] met the criteria for inclusion, leaving us with the two studies [38,39] featured in that review and the results reported above for the ZARADEMP cohort.

#### 3.2.2. Description of Included Studies

The three included studies comprised a total of 31,948 participants. Study details are presented in Table 3. Two were conducted in Europe ([38] and ZARADEMP) and one in Australia [39]. Two studies featured both women and men ([39] and ZARADEMP), and one only men [38].

All studies used clinical criteria to define VaD. However, the way anxiety was assessed differed between them. These included clinical criteria to define anxiety cases [39], a diagnostic interview (ZARADEMP), and a questionnaire which focused on either the presence of anxiety as personality trait or anxiety symptoms [38].

Follow-up periods ranged from 7.5 (ZARADEMP) to 20.4 years [39]. All studies adjusted for several covariates, with all including age and education in the analyses. The three studies reported adjusted ORs as an estimate for the link between anxiety and incident VaD. ORs ranged from 1.41 (95% CI: 0.75–2.68) (ZARADEMP) to 2.79 (95% CI: 0.60–13.06) [38].

#### 3.2.3. Risk of Bias

ZARADEMP and the Australian study [39] were considered to have a low risk of bias (7–9 from 9 points), whereas the study featuring only men [38] did not represent the broader population and had a quality score of 6, indicating a medium risk of bias (Table 3, Supplementary Tables S3 and S4).

#### 3.2.4. Meta-Analysis of VaD Incidence

**Figure 1:**
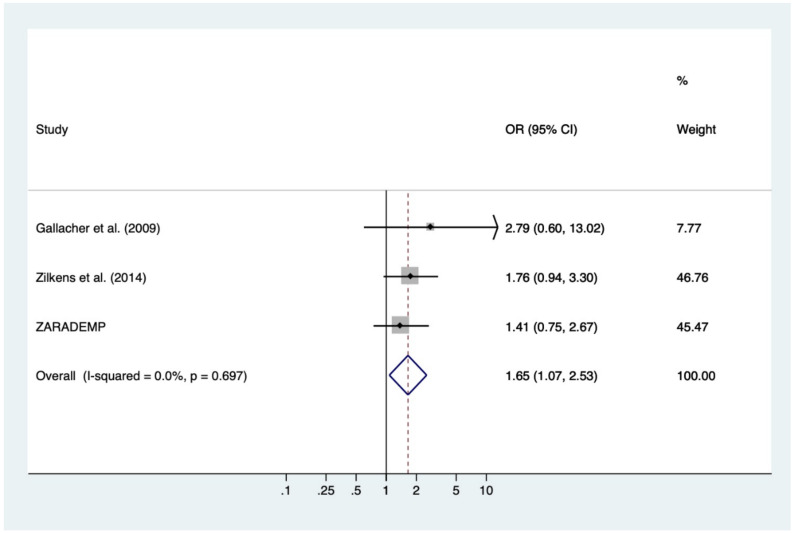
Forest plot showing individual and combined estimates for the risk of VaD associated with anxiety.

There was no evidence of heterogeneity among the three included studies, with *I^2^* = 0% (95% CI: 0–90%) and a nonsignificant Hedges test result (Q = 0.72; degrees of freedom (df) = 2; *p* = 0.697). Therefore, a fixed-effects model was carried out. Figure 1 shows the estimates of each study and the overall estimate for the association between anxiety and risk of incident VaD. All three OR estimates were greater than 1 (though none statistically significant), and the pooled OR was 1.65 (95% CI: 1.07–2.53) and significant (*p* = 0.022). This suggests that subjects with anxiety have 1.65 times higher risk of VaD than subjects without anxiety. The effect size was almost “medium” (Cohen’s d = 0.28; 95% CI: 0.04–0.51).

#### 3.2.5. Sensitivity Analysis

After excluding each study one-by-one from the analysis, the pooled OR did not substantially change; it varied between 1.56 (95% CI: 0.86–2.80) ([39] excluded) and 1.88 (95% CI: 1.05–3.36) (ZARADEMP excluded). This indicates that no single study had a disproportional impact on the overall OR (Supplementary Figure S2).

#### 3.2.6. Risk of Publication Bias

Publication bias was indicated by a fail-safe N equal to 1, suggesting that only one study with a null result is necessary to reduce the observed overall OR to nonsignificance.

## 4. Discussion

### 4.1. Main Findings

The present meta-analysis (MA) of three cohort studies found that the odds of developing VaD are 1.65 higher given the presence of anxiety at baseline.

### 4.2. Comparison with Previous Studies

Our finding of a pooled OR of 1.65 (95% CI: 1.07–2.53; *p* = 0.022) for the association between anxiety and risk of VaD is lower than the OR of 1.88 (95% CI, 1.05–3.36, *p* = 0.6) reported by a previous MA that featured one less study [8]. Our MA included a new cohort study, which extends evidence of causal links between the outcome (VaD) and the exposure (anxiety) in older people [40].

### 4.3. Potential Underlying Biological Mechanisms

There are several mechanisms that might explain a relationship between anxiety and an increased risk of VaD. Cerebrovascular lesions that affect critical brain regions are implicated in VaD [41] and could be caused by systemic, cardiac or primary cerebrovascular etiologies [42]. Anxiety, in turn, has been related to an increased risk of cardio- and cerebrovascular events (including stroke, coronary heart disease, heart failure, and cardiovascular death) [43,44]. There are several hypotheses about the underlying mechanisms linking anxiety disorders and cardiac diseases, including both lifestyles and physiological mechanism. People with anxiety are more likely to engage in unhealthy behaviors, including increased dietary cholesterol intake, elevated total energy intake, sedentary lifestyle, decreased physical activity, and substance abuse [45]. Anxiety has also been linked to increased inflammatory markers, changes in vascular endothelium, and greater platelet aggregation that can promote cardiac events [44,45]. In a systematic review, anxiety was found to be associated with significant reductions in heart rate variability, and this effect was independent from medication use and other medical and psychiatric comorbidity [46]. Altogether, these behavioral and physiological mechanisms linked to anxiety disorders might increase the risk of cardiovascular events [45]. In addition, anxiety is linked to elevated levels of glucocorticoids [47], which in turn might increase the risk of cardiovascular diseases [48].

However, one could not exclude a potential survival bias of those people who experience anxiety during the lifespan. The higher risk of cardiovascular events and premature cardiovascular-related death among people suffering from anxiety could then significantly decrease life expectancy of people suffering from anxiety disorders [49].

On the other hand, anxiety has been suggested to induce accelerated aging [50], which is an important risk factor for dementia. Moreover, anxiety could promote negative neuroplasticity [51] and facilitate a reduction of cognitive reserve. Additionally, recent studies indicate that anxiety could be related to increased gut permeability [52] and changes in the composition of gut microbiota [53], which may modulate cognitive functioning [54]. Future studies are needed to explore these potential biological mechanisms linking anxiety and risk of VaD.

While it is not yet clear whether anxiety is a risk factor or a prodromal syndrome of VaD, a recent MA [5] and systematic review [55] have supported anxiety being an independent risk factor for all-cause dementia. Similarly, our results seem to suggest that anxiety is a risk factor for VaD rather than a prodrome, given the relatively long intervals between the assessments of anxiety and VaD. Additionally, the studies we investigated excluded participants with dementia or MCI at baseline, thus minimizing the possibility of including a “subsyndromal” dementia.

### 4.4. Strengths and Limitations

Our study has several strengths. As an MA of all available studies of anxiety and risk of VaD, it has greater power to detect differences compared to the individual studies, none of which found a significant effect. The large sample size of the studies included in this MA can help to reduce risks associated with small study effects. Additionally, their follow-up periods were long enough for a sufficient number of incident dementia cases to be observed. Finally, this MA was based on adjusted OR estimations, thus providing a more accurate effect estimation.

There are also some limitations that should be considered when interpreting our results. First, only three studies were included in our MA, but this does not preclude validity [56,57]. Second, one of the studies only included men. However, sensitivity analysis showed that results did not differ when this study was excluded. Third, the number of incident cases reported in the ZARADEMP study was very low, which might in turn have resulted in a lack of positive significant results. However, similar low-incident cases of vascular dementia have been reported by previous population-based studies conducted in developed countries [58,59]. This is in concordance with an observed decrement of the incidence of vascular dementia over the last decade, probably linked to an improvement in vascular care [7]. Fourth, we used the GSM–AGECAT criteria to identify cases and subcases of anxiety in the ZARADEMP study, instead of using DSM/ICD criteria, which are widely used. However, the reliability and validity of this tool in older samples has been previously reported [60], which confers confidence in the diagnostic criteria. Fifth, there was some evidence of publication bias. Finally, methods used for assessing anxiety are different across the studies included in this MA. The use of different diagnostic methods could make comparability between studies difficult. However, since they all use validated methods of anxiety diagnosis and provide measures of association between anxiety and VaD, providing a summary measure might help to elucidate this complex relationship. More prospective studies, with larger samples and using standardized methods to corroborate this, are needed.

### 4.5. Clinical and Public Health Implications

Our finding that older individuals with anxiety are 1.65 at a higher risk of VaD has important clinical and public health implications because anxiety is a common, yet treatable mental health condition [4]. Anxiety negatively impacts general health and is related to lower levels of physical activity, higher social isolation, and poor dietary habits [44], which are themselves modifiable risk factors for dementia [61]. Thus, if anxiety is a risk factor for VaD rather than a prodrome, treating anxiety might help to prevent VaD. However, the diagnosis and assessment of late-life anxiety can be problematic because some anxiety symptoms may be attributed to the normal aging process (e.g., older adults and clinicians often view anxiety, fear, and avoidance as normal given aging circumstances) or are frequently comorbid with other medical and mental conditions (e.g., including muscle tension and difficulties related to sleep) [62].

Further, there is an unclear relationship between the use of benzodiazepines and the risk of dementia. Some studies have found a significant association between benzodiazepine and an increased risk of dementia [63,64], but other studies have not [65,66]. Additionally, very limited and inconsistent evidence exists on the link between benzodiazepine use and risk of dementia subtypes, such as AD or VaD [67]. These inconsistencies might be explained in the light of different timing of use, or due to reverse causation, because benzodiazepines are often prescribed to treat symptoms that are prodromes of dementia, such as anxiety or insomnia. Future research is needed to clarify if anxiolytic medication increases the risk of all-cause dementia and subtypes including VaD.

## 5. Conclusions

Our findings indicate that anxiety seems to significantly increase the risk of developing VaD. Considering the high prevalence of anxiety among older adults, interventions to prevent or treat anxiety might help to reduce the incidence and prevalence of VaD, as well as its heavy personal and societal burden. However, future research needs to still clarify the role of anxiety, that is, whether it is a genuine risk factor of VaD rather than a prodrome or a marker of the disease.

## Figures and Tables

**Table 1:** Sociodemographic characteristics, medical risk factors, health status, depression, and cognitive status of the participants in the Zaragoza Dementia and Depression (ZARADEMP) study.

Variable	No Incident VaD (*n* = 4013)	Incident VaD (*n* = 44)	*p*
Age (years)	72.0 (9.1)	78.7 (7.6)	<0.001
Women, n (%)	2208 (55.0)	21 (47.7)	0.333
Education			0.284
Primary school, n (%)	2984 (75)	35 (79.5)	
High school or higher, n (%)	686 (17.2)	4 (9.1)	
Marital status			0.047
Married/in couple, n (%)	2504 (62.6)	24 (54.5)	
Divorced/separated/widowed, n (%)	1133 (28.3)	19 (43.2)	
Living alone, n (%)	694 (17.3)	7 (15.9)	0.809
Body mass index (kg/m^2^)	26.7 (6.0)	27.4 (4.4)	0.469
Hypertension, n (%)	2711 (67.7)	36 (81.8)	0.046
Diabetes, n (%)	497 (12.5)	4 (9.1)	0.497
Vascular disease, n (%)	450 (11.2)	9 (20.9)	0.046
Health status (‘not good’), n (%)	2079 (51.9)	22 (50.0)	0.805
Depression, n (%)	452 (11.3)	3 (6.8)	0.353
Anxiety (‘Subcase/case’), n (%)	1716 (42.8)	20 (45.5)	0.719
MMSE score	27.2 (2.5)	25.8 (2.5)	<0.001

Notes: Data are mean (SD), unless otherwise indicated. VaD: Vascular dementia. MMSE = Mini-Mental State Examination.

**Table 2:** Risk of VaD associated with anxiety using logistic regression.

	Model 1		Model 2	
	OR (95% CI)^a^	*p*	OR (95% CI)^a^	*p*
Anxiety status at baseline				
Noncase	1	−	1	−
Subcase/Case	1.16 (0.63–2.15)	0.624	1.41 (0.71–2.68)	0.288

Notes: OR: Odds Ratio. ^a^ Reported OR of VaD is related to non-cases. Model 1 included anxiety status, as well as sociodemographic characteristics (sex, age, educational level, marital status, and living alone). Model 2 included everything in Model 1 plus medical risk factors (vascular disease, hypertension, obesity and diabetes), health status, depression, cognitive status at baseline (MMSE score), and follow-up time.

**Table 3:** Characteristics of included studies.

Authors, Year (no. of Participants)	Country (Study Design)	Follow-up Period (Years), Mean (SD)	Age (Years), Mean (SD)	Females, n (%)	Anxiety Measure; Prevalent Cases, n (%)	Vascular Dementia Criteria (no. of Incident Cases)	Risk Estimates (95% CI)	Covariates	Quality Score
Gallacher et al. [38] (n = 755)	United Kingdom (Prospective)	17.3 (1.3)	NR	0 (0)	STAI (cut-off: >35); NR	DSM-IV, NINDS-AIREN) criteria (NR)	OR: 2.79 (0.60–13.06)	Age, vascular risk factors, GHQ and NART	6
Zilkens et al. [39] (n = 27,136)	Australia (Retrospective: case-control)	20.4 (10.4)	78.7 (4.7)	15,359 (56.6)	ICD-10; 379 (2.8)	ICD-8; 9; 9-CM, 10-AM (1280)	OR: 1.76 (0.94–3.30)	Diabetes, heart disease, cerebrovascular disease and smoking risk factors.	7
ZARADEMP (n = 4057)	Spain (Prospective)	7.5 (4.2)	72.1 (9.1)	2229 (54.9)	Cases and subcases of anxiety using GMS–AGECAT; 1736 (42.8)	DSM-IV and Hachinski scale (44)	OR: 1.41 (0.75–2.68)	Sociodemographic characteristics (age, sex, education, marital status and living alone), medical risk factors (body mass index, previous vascular disease, hypertension and diabetes), health status, depression and cognitive status at baseline (MMSE).	8

Abbreviations in the table: DSM-IV: Diagnostic and Statistical Manual, Fourth Edition; GHQ: General health questionnaire; GMS–AGECAT: Geriatric Examination for computer-assisted taxonomy; NART: National adult reading test; NR: Not reported; OR: Odds ratio; SD: Standard deviation; STAI: State-Trait Anxiety Inventory; y: Years.

## Supplementary Materials

The following are available online at https://www.mdpi.com/2077-0383/9/5/1368/s1, Figure S1: Flowchart of literature search and study selection, Figure S2: Sensitivity analysis, Table S1: PRISMA checklist, Table S2: Search strategy, Table S3: Quality assessment of cohort studies in the meta-analysis using the Newcastle–Ottawa scale (NOS), Table S4: Quality assessment of case-control studies in the meta-analysis using the Newcastle–Ottawa scale (NOS).
