# Supplementary material for: Association between Anxiety and Vascular Dementia Risk: New Evidence and an Updated Meta-Analysis
Source: J Clin Med. 2020 May 6;9(5):1368. doi: 10.3390/jcm9051368 (PMC7291213; doi:10.3390/jcm9051368)
Supplement: Supplementary file 1 [file jcm-09-01368-s001.pdf]

Supplementary Materials: Association between Anxiety and Vascular Dementia Risk: New Evidence and An Updated Meta-Analysis

Javier Santabárbara, Darren M. Lipnicki, Beatriz Olaya, Beatriz Villagrasa, Patricia Gracia-García, Juan Bueno-Notivol, Antonio Lobo and Raúl López-Antón

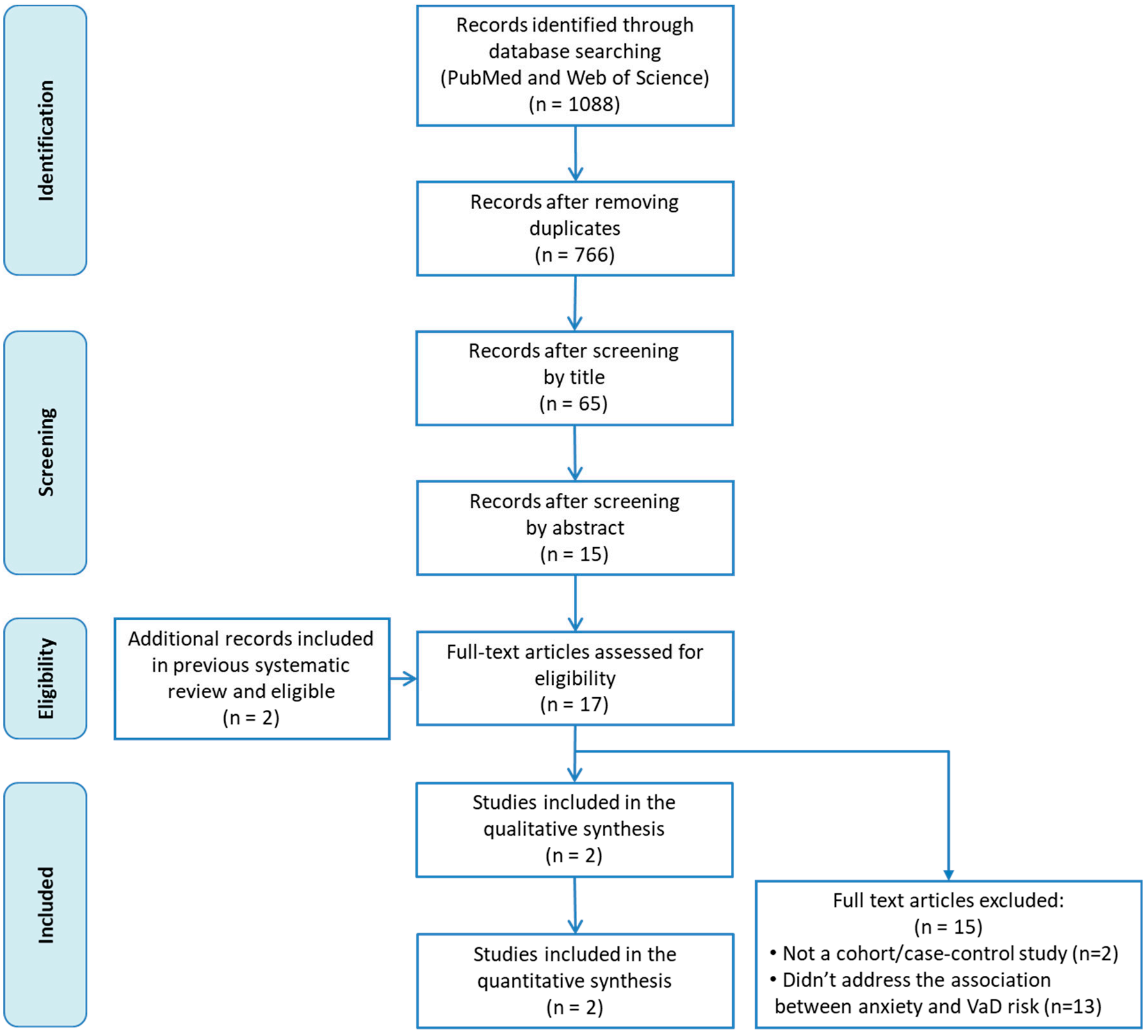

Figure S1. Flowchart of literature search and study selection.

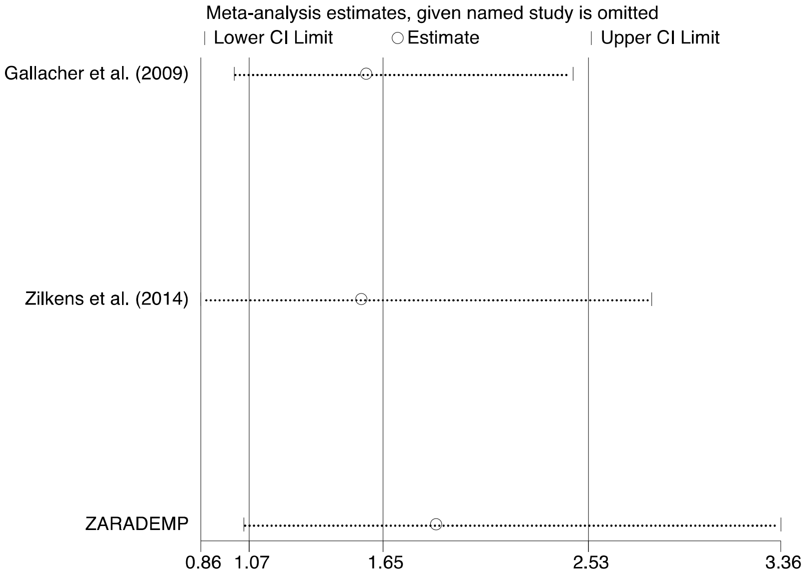

Figure S2. Sensitivity analysis.

Table S1. PRISMA checklist.

Section/topic	#	Checklist item	Reported on page #
		**Title**	
Title	1	Identify the report as a systematic review, meta-analysis, or both.	1
		**Abstract**	
Structured summary	2	Provide a structured summary including, as applicable: background; objectives; data sources; study eligibility criteria, participants, and interventions; study appraisal and synthesis methods; results; limitations; conclusions and implications of key findings; systematic review registration number.	2
		**Introduction**	
Rationale	3	Describe the rationale for the review in the context of what is already known.	3
Objectives	4	Provide an explicit statement of questions being addressed with reference to participants, interventions, comparisons, outcomes, and study design (PICOS).	3
		**Methods**	
Protocol and registration	5	Indicate if a review protocol exists, if and where it can be accessed (e.g., Web address), and, if available, provide registration information including registration number.	-
Eligibility criteria	6	Specify study characteristics (e.g., PICOS, length of follow-up) and report characteristics (e.g., years considered, language, publication status) used as criteria for eligibility, giving rationale.	5
Information sources	7	Describe all information sources (e.g., databases with dates of coverage, contact with study authors to identify additional studies) in the search and date last searched.	5
Search	8	Present full electronic search strategy for at least one database, including any limits used, such that it could be repeated.	5
Study selection	9	State the process for selecting studies (i.e., screening, eligibility, included in systematic review, and, if applicable, included in the meta-analysis).	5
Data collection process	10	Describe method of data extraction from reports (e.g., piloted forms, independently, in duplicate) and any processes for obtaining and confirming data from investigators.	5
Data items	11	List and define all variables for which data were sought (e.g., PICOS, funding sources) and any assumptions and simplifications made.	5
Risk of bias in individual studies	12	Describe methods used for assessing risk of bias of individual studies (including specification of whether this was done at the study or outcome level), and how this information is to be used in any data synthesis.	5
Summary measures	13	State the principal summary measures (e.g., risk ratio, difference in means).	5
Synthesis of results	14	Describe the methods of handling data and combining results of studies, if done, including measures of consistency (e.g., I^2) for each meta-analysis.	5
Risk of bias across studies	15	Specify any assessment of risk of bias that may affect the cumulative evidence (e.g., publication bias, selective reporting within studies).	6
Additional analyses	16	Describe methods of additional analyses (e.g., sensitivity or subgroup analyses, meta-regression), if done, indicating which were pre-specified.	6

**Results**			
Study selection	17	Give numbers of studies screened, assessed for eligibility, and included in the review, with reasons for exclusions at each stage, ideally with a flow diagram.	6, Supplementary Figure 1
Study characteristics	18	For each study, present characteristics for which data were extracted (e.g., study size, PICOS, follow-up period) and provide the citations.	7
Risk of bias within studies	19	Present data on risk of bias of each study and, if available, any outcome level assessment (see item 12).	Table 2
Results of individual studies	20	For all outcomes considered (benefits or harms), present, for each study: (a) simple summary data for each intervention group (b) effect estimates and confidence intervals, ideally with a forest plot.	7, Table 2, Figure 1
Synthesis of results	21	Present results of each meta-analysis done, including confidence intervals and measures of consistency.	7
Risk of bias across studies	22	Present results of any assessment of risk of bias across studies (see Item 15).	7, Table 3, Supplementary table 3 and Supplementary table 4
Additional analysis	23	Give results of additional analyses, if done (e.g., sensitivity or subgroup analyses, meta-regression [see Item 16]).	7, Supplementary Figure 2
**Discussion**			
Summary of evidence	24	Summarize the main findings including the strength of evidence for each main outcome; consider their relevance to key groups (e.g., healthcare providers, users, and policy makers).	8
Limitations	25	Discuss limitations at study and outcome level (e.g., risk of bias), and at review-level (e.g., incomplete retrieval of identified research, reporting bias).	8–9
Conclusions	26	Provide a general interpretation of the results in the context of other evidence, and implications for future research.	9
**Funding**			
Funding	27	Describe sources of funding for the systematic review and other support (e.g., supply of data); role of funders for the systematic review.	9

Adapt from [11]: Moher D, Liberati A, Tetzlaff J, Altman DG, The PRISMA Group (2009). Preferred Reporting Items for Systematic Reviews and Meta-Analyses: The PRISMA Statement. PLoS Med 6(7): e1000097. doi:10.1371/journal.pmed1000097. For more information, visit: www.prisma-statement.org.

Table S2. Search strategy.

PubMed: (“anxiety”[Mesh] OR "Phobic Disorders"[Mesh] OR "Anxiety Disorders"[Mesh] OR “anxiety”[All Fields] OR “neuropsychiatric symptoms”[All Fields]) **AND** (“dementia”[Mesh] OR “dementia”[All Fields] OR "Dementia, Vascular"[Mesh] OR "vascular dementia"[All Fields]) **AND** ("Cohort Studies"[Mesh] OR ("cohort"[All Fields] AND "stud*"[All Fields]) OR "cohort stud*"[All Fields] OR "Incidence"[Mesh] OR “incidence”[All Fields] OR "epidemiology"[Subheading] OR "Epidemiology"[Mesh] OR "epidemiology"[All Fields] OR “risk”[All Fields] OR "Case-Control Studies"[Mesh] OR “case-control” [All Fields]) **AND** English[lang] **AND** ("2018/01/12"[PDAT] : "2019/10/31” [PDAT]).

WoS: (AB=(“anxiety” OR "Phobic Disorder*" OR "Anxiety Disorder*" OR “neuropsychiatric symptom*”) *OR* TI=(“anxiety” OR "Phobic Disorder*" OR "Anxiety Disorder*" OR “neuropsychiatric symptom*”) *OR* TS=(“anxiety” OR "Phobic Disorder*" OR "Anxiety Disorder*" OR “neuropsychiatric symptom*”)) ***AND*** (AB=(“dementia” OR "vascular dementia") *OR* TI=(“dementia” OR "vascular dementia") *OR* TS=(“dementia” OR "vascular dementia")) ***AND*** (AB=(“cohort” OR "incidence" OR "epidemiology" OR “risk” OR “case-control” OR case control) *OR* TI=(“cohort” OR "incidence" OR "epidemiology" OR “risk” OR “case-control” OR case control) *OR* TS=(“cohort” OR "incidence" OR "epidemiology" OR “risk” OR “case-control” OR case control)) (Limited to years 2018 and 2019 and articles in English).

Table S3. Quality assessment of cohort studies in the meta-analysis using the Newcastle–Ottawa scale (NOS).

Quality assessment criteria	Acceptable (*)	Gallacher et al. [35]	ZARADEMP
**Selection**			
Representativeness of exposed cohort	Representative of average older in community (age/sex/being at risk of disease)	–	*
Selection of the non-exposed cohort	Drawn from same community as exposed cohort	*	*
Ascertainment of exposure	Secured records or structured interview	*	*
Demonstration that outcome of interest was not present at start of study	Only incident cases of vascular dementia	–	*
**Comparability**			
Comparability of cohorts on the basis of the design or analysis	Study controls for age and sex	–	*
Comparability of cohorts on the basis of the design or analysis	Study controls for any additional factor (education attainment, depression or vascular risk factor)	*	*
**Outcome**			
Assessment of outcome	Independent blind assessment, record linkage	*	–
Was follow-up long enough for outcome to occur	Follow-up ≥ 10 years	*	*
Adequacy of follow-up of cohorts	Complete follow-up (all subjects accounted for or subjects lost to follow-up unlikely to introduce bias)	*	*
**Overall Quality Score (Maximum = 9)**		**6**	**8**

* Indicates that that particular quality criteria is acceptable for a given study.

Table S4. Quality assessment of case-control studies in the meta-analysis using the Newcastle–Ottawa scale (NOS).

Quality assessment criteria	Acceptable (*)	Zilkens et al. [36]
**Selection**		
Is the case definition adequate?	Independent validation	–
Representativeness of the cases	Consecutive or obviously representative series of	*

	cases	
Selection of controls	Community controls	*
Definition of controls	No history of disease (endpoint)	*
	**Comparability**	
Comparability of cases and controls on the basis of the design or analysis	Study controls for age/sex	*
Comparability of cases and controls on the basis of the design or analysis	Study controls for any additional factor (education attainment, depression or vascular risk factor)	*
	**Outcome**	
Ascertainment of exposure	Secure records (e.g., clinical records) or structured interview where blind to case/control status	*
Same method of ascertainment for cases and controls	Yes	*
Non-response rate	Same rate for both groups	–
**Overall Quality Score (Maximum = 9)**		**7**

* Indicates that that particular quality criteria is acceptable for a given study.
